# Optimizing radiomics for prostate cancer diagnosis: feature selection strategies, machine learning classifiers, and MRI sequences

**DOI:** 10.1186/s13244-024-01783-9

**Published:** 2024-11-04

**Authors:** Eugenia Mylona, Dimitrios I. Zaridis, Charalampos Ν. Kalantzopoulos, Nikolaos S. Tachos, Daniele Regge, Nikolaos Papanikolaou, Manolis Tsiknakis, Kostas Marias, ProCAncer-I Consortium, Eugenia Mylona, Dimitris Zaridis, Charalampos Kalantzopoulos, Nikolaos S. Tachos, Daniele Regge, Nikolaos Papanikolaou, Manolis Tsiknakis, Kostas Marias, Dimitris Fotiadis, Stelios Sfakianakis, Varvara Kalokyri, Eleftherios Trivizakis, Grigorios Kalliatakis, Avtantil Dimitriadis, José Guilherme de Almeida, Ana Castro Verde, Ana Carolina Rodrigues, Nuno Rodrigues, Miguel Chambel, Henkjan Huisman, Maarten de Rooij, Anindo Saha, Jasper J. Twilt, Jurgen Futterer, Luis Martí-Bonmatí, Leonor Cerdá-Alberich, Gloria Ribas, Silvia Navarro, Manuel Marfil, Emanuele Neri, Giacomo Aringhieri, Lorenzo Tumminello, Vincenzo Mendola, nan, Deniz Akata, Mustafa Özmen, Ali Devrim Karaosmanoglu, Firat Atak, Musturay Karcaaltincaba, Joan C. Vilanova, Jurgita Usinskiene, Ruta Briediene, Audrius Untanas, Kristina Slidevska, Katsaros Vasilis, Georgiou Georgios, Dow-Mu Koh, Robby Emsley, Sharon Vit, Ana Ribeiro, Simon Doran, Tiaan Jacobs, Gracián García-Martí, Valentina Giannini, Simone Mazzetti, Giovanni Cappello, Giovanni Maimone, Valentina Napolitano, Sara Colantonio, Maria Antonietta Pascali, Eva Pachetti, Giulio del Corso, Danila Germanese, Andrea Berti, Gianluca Carloni, Jayashree Kalpathy-Cramer, Christopher Bridge, Joao Correia, Walter Hernandez, Zoi Giavri, Christos Pollalis, Dimitrios Agraniotis, Ana Jiménez Pastor, Jose Munuera Mora, Clara Saillant, Theresa Henne, Rodessa Marquez, Dimitrios I. Fotiadis

**Affiliations:** 1 Biomedical Research Institute, FORTH, GR 45110 Ioannina, Greece; 2 Unit of Medical Technology Intelligent Information Systems, University of Ioannina, Ioannina, Greece; 3 Biomedical Engineering Laboratory, School of Electrical & Computer Engineering, National Technical University of Athens, Athens, Greece; 4 Department of Radiology, Candiolo Cancer Institute, FPO-IRCCS, Candiolo, Italy; 5 Computational Clinical Imaging Group, Champalimaud Foundation, Lisboa, Portugal; 6 Computational Biomedicine Laboratory, Institute of Computer Science, FORTH, GR 70013 Heraklion, Greece; 7 Department of Electrical and Computer Engineering, Hellenic Mediterranean University, GR 71004 Heraklion, Greece; 8 FORTH—Institute of Molecular Biology and Biotechnology (FORTH-IMBB/BR), Heraklion, Greece; 9 Candiolo Cancer Institute, FPO-IRCCS, Candiolo, Italy; 10 Champalimaud Foundation, Lisboa, Portugal; 11 Radboud, Nijmegen, Netherlands; 12 HULAFE—Biomedical Imaging Research Group, Instituto de Investigación Sanitaria La Fe, Valencia, Spain; 13 Medical Imaging Department, Hospital Universitari i Politècnic La Fe, Valencia, Spain; 14 Academic Radiology, Department of Translational Research, University of Pisa, Pisa, Italy; 15 Institut Paoli-Calmettes, Marseille, France; 16 Department of Radiology, Hacettepe, Ankara, Turkey; 17 Department of Radiology (IDI), Institute of Biomedical Research of Girona Dr. Josep Trueta (IDIBGI), Girona, Spain; 18 National Cancer Institute, Vilnius, Lithuania; 19 General Anti-Cancer and Oncological Hospital of Athens, Athens, Greece; 20 Radiology & AI Research Hub, The Royal Marsden NHS Foundation Trust, London, UK; 21 Division of Radiotherapy and Imaging, The Institute of Cancer Research, London, UK; 22 Quirónsalud Hospital/CIBERSAM, Valencia, Spain; 23 Institute of Information Science and Technologies of the National Reserch Council of Italy, Pisa, Italy; 24 Mass General Hospital, Boston, MA USA; 25 B3D, London, UK; 26 Advantis, Athens, Greece; 27 Quibim S.L., Valencia, Spain; 28 Univie, Vienna, Austria

**Keywords:** Radiomics, Prostate cancer, Machine learning, MRI

## Abstract

**Objectives:**

Radiomics-based analyses encompass multiple steps, leading to ambiguity regarding the optimal approaches for enhancing model performance. This study compares the effect of several feature selection methods, machine learning (ML) classifiers, and sources of radiomic features, on models’ performance for the diagnosis of clinically significant prostate cancer (csPCa) from bi-parametric MRI.

**Methods:**

Two multi-centric datasets, with 465 and 204 patients each, were used to extract 1246 radiomic features per patient and MRI sequence. Ten feature selection methods, such as Boruta, mRMRe, ReliefF, recursive feature elimination (RFE), random forest (RF) variable importance, L1-lasso, etc., four ML classifiers, namely SVM, RF, LASSO, and boosted generalized linear model (GLM), and three sets of radiomics features, derived from T2w images, ADC maps, and their combination, were used to develop predictive models of csPCa. Their performance was evaluated in a nested cross-validation and externally, using seven performance metrics.

**Results:**

In total, 480 models were developed. In nested cross-validation, the best model combined Boruta with Boosted GLM (AUC = 0.71, F1 = 0.76). In external validation, the best model combined L1-lasso with boosted GLM (AUC = 0.71, F1 = 0.47). Overall, Boruta, RFE, L1-lasso, and RF variable importance were the top-performing feature selection methods, while the choice of ML classifier didn’t significantly affect the results. The ADC-derived features showed the highest discriminatory power with T2w-derived features being less informative, while their combination did not lead to improved performance.

**Conclusion:**

The choice of feature selection method and the source of radiomic features have a profound effect on the models’ performance for csPCa diagnosis.

**Critical relevance statement:**

This work may guide future radiomic research, paving the way for the development of more effective and reliable radiomic models; not only for advancing prostate cancer diagnostic strategies, but also for informing broader applications of radiomics in different medical contexts.

**Key Points:**

- Radiomics is a growing field that can still be optimized.
- Feature selection method impacts radiomics models’ performance more than ML algorithms.
- Best feature selection methods: RFE, LASSO, RF, and Boruta.
- ADC-derived radiomic features yield more robust models compared to T2w-derived radiomic features.

**Graphical Abstract:**

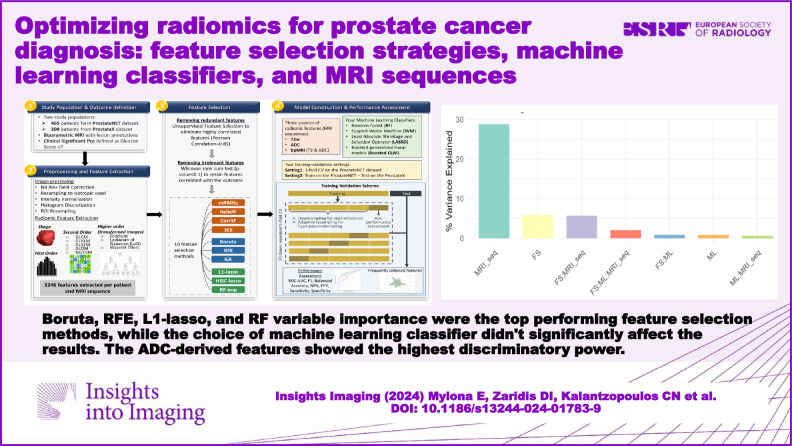

## Introduction

Prostate cancer (PCa) is a prevalent male disease and early diagnosis is the cornerstone of effective treatment, with the estimated 10-year survival rate reaching 99% [1]. Although a definite diagnosis of PCa is possible only through biopsy, magnetic resonance imaging (MRI) plays a pivotal role in the preliminary assessment and localization of suspicious areas [2].

Radiomics leverages the capabilities of artificial intelligence (AI) to harness non-invasive quantitative biomarkers extracted from medical images, linked to tumor heterogeneity and biological characteristics, aiding in the detection, diagnosis, and characterization of various diseases, including PCa [3–7]. A radiomics analysis comprises of several methodically interconnected steps, including image acquisition, region of interest (ROI) delineation, extraction of hundreds to thousands of radiomic features, feature selection, and model development [8].

Feature selection plays a pivotal role in the reliability and generalizability of radiomics workflows [9]. This is because redundancy and multicollinearity among the variables can have a detrimental impact on machine learning (ML) models, leading to misleading outcomes, overfitting, and reduced interpretability [10]. Prior efforts to address issues related to excessive feature dimensions, overfitting, and improving the predictive performance of ML classifiers, have focused on standardizing imaging biomarkers and addressing radiomic feature reproducibility and stability. Despite numerous advancements, there remains a critical gap in rigorously evaluating the performance of various feature selection methods and ML classifiers in radiomics, particularly in the context of PCa diagnosis [11].

Various feature selection methods have been proposed to reduce large radiomic datasets into a reasonable number of features that sufficiently describe the most relevant and predictive imaging characteristics for the classification task in question [12, 13]. The feature selection methods are divided into three methodological categories, including filter, wrapper, and embedded methods, each offering distinct approaches to identifying the most informative features while mitigating issues such as overfitting and dimensionality reduction [14]. Different feature selection methods may identify different features as relevant, and the choice of method can affect the performance of the radiomics model [13].

Several studies have also investigated the effect of algorithm choice on radiomics performance, and it has been found that model performances may vary greatly [15]. While tree-based methods, predominantly random forest (RF), tend to perform best, some studies have concluded the superiority of support vector machine (SVM) and linear models, as well [16–20]. Nevertheless, the impact of the choice of ML on radiomics models for the diagnosis of PCa remains elusive.

The objective of this study was to comprehensively assess the impact of several commonly used feature selection methods and ML classifiers, as well as biparametric MRI (bpMRI) sequences, in the context of radiomics-based PCa diagnosis, using different validation settings. While prior studies have explored individual aspects of radiomic analysis [16, 21–26], a comprehensive comparison of diverse feature selection techniques and predictive models has been lacking. This study aims to fill this gap by systematically evaluating these methods to enhance the performance and reliability of radiomics models in PCa research.

## Methods

**Fig. 1:**
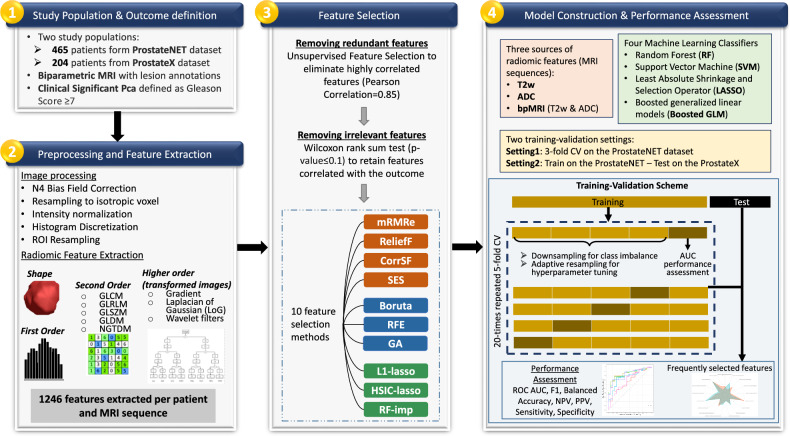
The workflow of the study in four steps

The workflow of the radiomics analysis, divided into four distinct steps, is presented in Fig. 1.

### Study population and outcome definition (step 1)

Two populations were used; the ProstateNET [27], which is a multi-centric, multi-vendor PCa dataset, and the publicly available ProstateX2 dataset [28]. The ProstateNET contains thousands of PCa multiparametric MRI (mpMRI) images acquired using different models of Siemens, Philips, and GE vendors, collected from 12 clinical centers across 8 European countries. For the purpose of our study, a total of 465 retrospectively collected patient data with manual lesion segmentations, acquired without an endorectal coil, were available. The ProstateX2 dataset consists of 204 patient mpMRI, acquired on two types of Siemens 3-T scanners, using a turbo spin echo sequence without an endorectal coil. The corresponding manually generated lesion segmentations [29, 30] are publicly available (https://github.com/rcuocolo/PROSTATEx_masks).

The clinical characteristics of the two datasets are provided in Table 1. Clinically significant prostate cancer (csPCa) was histologically determined through a biopsy or prostatectomy, and was defined as Gleason grade ≥ 2, based on the European Association of Urology guidelines, which corresponds to a Gleason score of ≥ 7 [31, 32]. Based on this definition, 74% of patients in the ProstateNET were diagnosed with csPCa, and 25% in the ProstateX2.

**Table 1:** Characteristics of the two study populations

Population characteristics	ProstateNET	ProstateX2
Number of patients	464	204
Age at diagnosis, (mean ± SD; years)	65.03 ± 7.9	63.47 ± 7.1
PSA total, (mean ± SD; ng/mL)	11.45 ± 18.89	13.62 ± 9.18
Lesion volume, (mL)	3.15 ± 6.8	1.42 ± 0.41
Patients with csPCa—Gleason score ≥ 7	74%	25%

### Preprocessing and feature extraction (step 2)

First, bias field correction was applied to all T2w images to compensate for intensity non-uniformities using the N4 Bias Field Correction algorithm [33] and the Python package SimpleITK (version 2.2.12.0.0). All the subsequent pre-processing steps, including resampling to isotropic voxel, the normalization of pixel intensity values, and discretization, were performed using functions embedded within the open-source PyRadiomics library (version 2.2.0) [34]. The exact configuration is given in Supplementary Table 1. All scans were resampled to 1 mm in-plane resolution and slice thickness using b-spline interpolation. The ROIs were also resampled to the apparent diffusion coefficient (ADC) maps. Intensity normalization was performed, scaling the values by a factor of 100. For histogram discretization, the absolute discretization approach (fixed bin size) was adopted as it tends to preserve a higher number of reproducible features for MRI compared to relative discretization [35]. The optimal bin width was defined so that the number of bins in each image histogram would range from 30 to 128 bins [36].

From each 3D ROI, representing the tumor volume, radiomic features were extracted from T2w scans and ADC maps. Feature extraction included shape, first order, gray level co-occurrence matrix, gray level run length matrix, gray level size zone matrix, gray level dependence matrix, and neighboring gray-tone difference matrix features. Beyond the original image domain, higher-order features were extracted from transformed images, after applying a Gradient filter, Laplacian of Gaussian filter with kernel sizes from 2 mm to 5 mm, and Wavelet filters with all the combinations of high- and low-pass filters on each image dimension. This process resulted in 1246 features being extracted per patient and MRI sequence.

### Feature selection (step 3)

Prior to feature selection, we removed highly correlated features and variables irrelevant to the outcome. Low variance features were excluded using a variance threshold of 0.01 and multicollinear features were eliminated using a Pearson correlation threshold of 0.85. If two variables had a correlation surpassing the threshold, we removed the one with the largest mean absolute correlation with other variables. Subsequently, supervised feature selection was applied solely to the training data to prevent data leakage. To retain only features correlated with the outcome, a Wilcoxon rank sum test was performed with a significance threshold of 0.1, allowing us to prioritize a subset of features for further feature selection. This approach significantly reduced the number of features (< 150 variables), facilitating a more exhaustive feature selection.

Some of the most commonly employed feature selection techniques were implemented, including four filter methods, three wrapper methods, and three embedded methods.

#### Filter methods

- Minimum redundancy maximum relevance ensemble (mRMRe): uses mutual information to select features correlated with the outcome (relevance) while minimizing the correlation between features (redundancy).
- ReliefF: evaluates feature importance based on instance learning, by assessing differences in feature values among different classes. A feature score is assigned to each feature based on differences between nearest neighbor instance pairs.
- Correlation-based feature selection with forward selection strategy (CorrSF): a forward selection strategy based on correlation coefficients to progressively include pertinent features while accounting for their interrelationships.
- Statistically equivalent multiple feature subsets (SES): a forward-backward feature selection, that assesses feature relevance through univariate association and conditional independence tests, ranking features based on statistical significance.

#### Wrapper methods

- Boruta: based on RF, features are selected by comparing their importance with that of shadow features (randomly permuted). The top-ranked features undergo *p*-value correction via the Benjamin Hochberg method [37], with those surpassing the threshold being selected.
- Recursive feature elimination (RFE): fits a model with all the features, and iteratively removes the weakest one. Herein, SVM was utilized for RFE-based feature selection.
- Genetic algorithm (GA): mimicking genetic evolution, it evaluates various feature subsets, evolves them over generations through genetic operations, and assesses their fitness using a predefined criterion.

#### Embedded methods

- L1-lasso: the least absolute shrinkage and selection operator (lasso) with L1-regularization is applied to linear models, penalizing the absolute coefficients to promote feature sparsity, driving some coefficients to zero [38].
- HSIC-lasso: integrates the Hilbert-Schmidt independence criterion (HSIC) [39] into the lasso framework. Unlike L1-lasso it measures the independence between the features and the outcome. It can be viewed as a convex variant of the mRMR feature selection algorithm.
- Random Forest variable importance (RF-imp): leverages the tree minimal depth methodology within an RF framework to evaluate the importance of features.

### Model construction and performance assessment (step 4)

Two experimental settings were considered for training and validating the predictive models [40]. In setting 1, a nested cross-validation (CV) on the ProstateNET dataset was performed with 3 outer folds, ensuring that the distribution of the target class and clinical sites was the same across folds. In setting 2, models were trained on the ProstateNET dataset and validated externally using the ProstateX2.

Regarding the imaging source of radiomics features, three scenarios were examined: (i) using T2w-derived features, (ii) using ADC-derived features, and (iii) combining T2w and ADC features (bpMRI).

For building the radiomics-based models, we selected four well-established and methodologically diverse ML classifiers, namely the RF, LASSO, and SVM with radial basis function, and boosted generalized linear models (Boosted GLM). Each classifier was trained with 20 times repeated 5-fold CV to tune the hyper-parameters. Downsampling was applied to balance the classes and parameter tuning was obtained through a grid search and adaptive resampling of the parameter grid.

Model performance was estimated based primarily on the area under the receiver operating characteristic curve (AUC) and the F1 score, but other metrics were also computed, including balanced accuracy (BA), negative predictive value (NPV), positive predictive value (PPV)/precision, sensitivity/recall, and specificity.

### Statistical analysis

Descriptive statistics were reported as mean values and standard deviation. Models’ performance in terms of ROC AUC was compared using DeLong’s test, and *p*-values less than 0.05 were considered significant. Due to multiple comparisons, Bonferroni correction was used to adjust the significance threshold and control the overall Type I error rate. The effect of different factors and their interactions on models’ performance was quantified through a multifactor analysis of variance. We tested the null hypothesis that there were no significant differences in the models’ AUC performance attributed to the factors under consideration. Specifically, we evaluated the main effects of feature selection, ML classifier, and MRI sequence, as well as their interactions. Additionally, the most commonly selected radiomic features across various feature selection methods and settings were identified, providing insights into the features that consistently contributed to predictive performance. Feature selection, predictive modeling, and statistical analyses were performed using R (version 4.3.0).

### Evaluation of radiomics research quality

To ensure credibility, reproducibility, and transparency of radiomics research, this study adhered to the CheckList for EvaluAtion of Radiomics Research (CLEAR) [41] reporting guidelines, and its quality was assessed using the METhodological RadiomICs Score (METRICS) [42]. In total, 44 out of 58 items in the CLEAR checklist were addressed (yes:44; no:10; and n/a: 4) and the METRICS quality score was “Excellent” (81.7%). Details of the CLEAR and METRICS scores are summarized in Supplementary Tables S2 and S3.

## Results

Given the different validation settings, MRI sequences, feature selection methods, and ML classifiers, the study resulted in a total of 480 radiomic models. Tables 2 and 3 show the average models’ performance (AUC and F1), using different feature selection methods, for settings 1 and 2, respectively. In both cases, the ADC-derived radiomic features resulted in a higher average performance compared to T2w radiomic features, while the combination of T2w and ADC features (bpMRI) did not yield any noticeable improvements. In setting 1, the mRMRe and RF-imp methods led to the highest average AUC (0.74 ± 0.04) and F1 (0.78 ± 0.04), respectively. In setting 2, the L1-lasso and RF-imp resulted in the best AUC (0.74 ± 0.04), while RFE produced the highest F1 (0.78 ± 0.04). Detailed performance assessment for each classifier and feature selection method is provided in Supplementary Figs. S1 and S2.

**Table 2:** Average AUC and F1-score achieved with each feature selection method in setting 1, stratified by MRI sequence

Feature selection	AUC			F1 score		
	T2w	ADC	bpMRI	T2w	ADC	bpMRI
Boruta	0.68 ± 0.04	0.71 ± 0.03	0.72 ± 0.03	0.74 ± 0.03	0.76 ± 0.03	0.77 ± 0.03
CorrSF	0.65 ± 0.03	0.72 ± 0.03	0.71 ± 0.02	0.72 ± 0.03	0.76 ± 0.04	0.74 ± 0.03
GA	0.66 ± 0.02	0.71 ± 0.04	0.67 ± 0.03	0.73 ± 0.03	0.76 ± 0.04	0.74 ± 0.02
HSIC-lasso	0.66 ± 0.04	0.71 ± 0.04	0.7 ± 0.03	0.73 ± 0.04	0.76 ± 0.05	0.75 ± 0.04
L1-lasso	0.68 ± 0.05	0.71 ± 0.06	0.69 ± 0.05	0.74 ± 0.05	0.77 ± 0.04	0.77 ± 0.02
mRMRe	0.62 ± 0.04	0.74 ± 0.04	0.72 ± 0.05	0.65 ± 0.04	0.76 ± 0.05	0.74 ± 0.04
SES	0.67 ± 0.06	0.7 ± 0.04	0.68 ± 0.05	0.74 ± 0.04	0.77 ± 0.04	0.77 ± 0.04
Relief	0.65 ± 0.05	0.7 ± 0.03	0.63 ± 0.03	0.74 ± 0.05	0.76 ± 0.04	0.76 ± 0.03
RF-imp	0.68 ± 0.04	0.72 ± 0.03	0.71 ± 0.02	0.74 ± 0.03	0.78 ± 0.04	0.76 ± 0.04
RFE	0.66 ± 0.04	0.72 ± 0.04	0.72 ± 0.02	0.73 ± 0.04	0.76 ± 0.04	0.77 ± 0.02

**Table 3:** Average AUC and F1-score achieved with each feature selection method in setting 2, stratified by MRI sequence

Feature selection	AUC			F1 score		
	T2w	ADC	bpMRI	T2w	ADC	bpMRI
Boruta	0.63 ± 0.01	0.7 ± 0.01	0.71 ± 0.01	0.42 ± 0.02	0.47 ± 0.02	0.46 ± 0.02
CorrSF	0.62 ± 0.01	0.71 ± 0.02	0.71 ± 0.02	0.42 ± 0.01	0.46 ± 0.01	0.45 ± 0.01
GA	0.62 ± 0.01	0.73 ± 0.01	0.65 ± 0.03	0.42 ± 0.02	0.46 ± 0.02	0.46 ± 0.02
HSIC-lasso	0.6 ± 0.02	0.71 ± 0.03	0.71 ± 0.02	0.42 ± 0.02	0.47 ± 0.01	0.47 ± 0.01
L1-lasso	0.65 ± 0.01	0.73 ± 0.02	0.71 ± 0.01	0.42 ± 0.02	0.48 ± 0.01	0.48 ± 0.02
mRMRe	0.63 ± 0	0.68 ± 0.03	0.68 ± 0.02	0.41 ± 0.01	0.48 ± 0.01	0.46 ± 0.01
SES	0.62 ± 0.01	0.68 ± 0.01	0.68 ± 0.01	0.41 ± 0.01	0.46 ± 0.01	0.47 ± 0
Relief	0.62 ± 0	0.66 ± 0.02	0.71 ± 0.02	0.42 ± 0.01	0.47 ± 0.02	0.47 ± 0.01
RF-imp	0.62 ± 0.01	0.7 ± 0.04	0.73 ± 0.02	0.4 ± 0.03	0.47 ± 0.01	0.49 ± 0.01
RFE	0.63 ± 0.02	0.71 ± 0.01	0.72 ± 0.02	0.44 ± 0.01	0.5 ± 0.02	0.41 ± 0

**Fig. 2:**
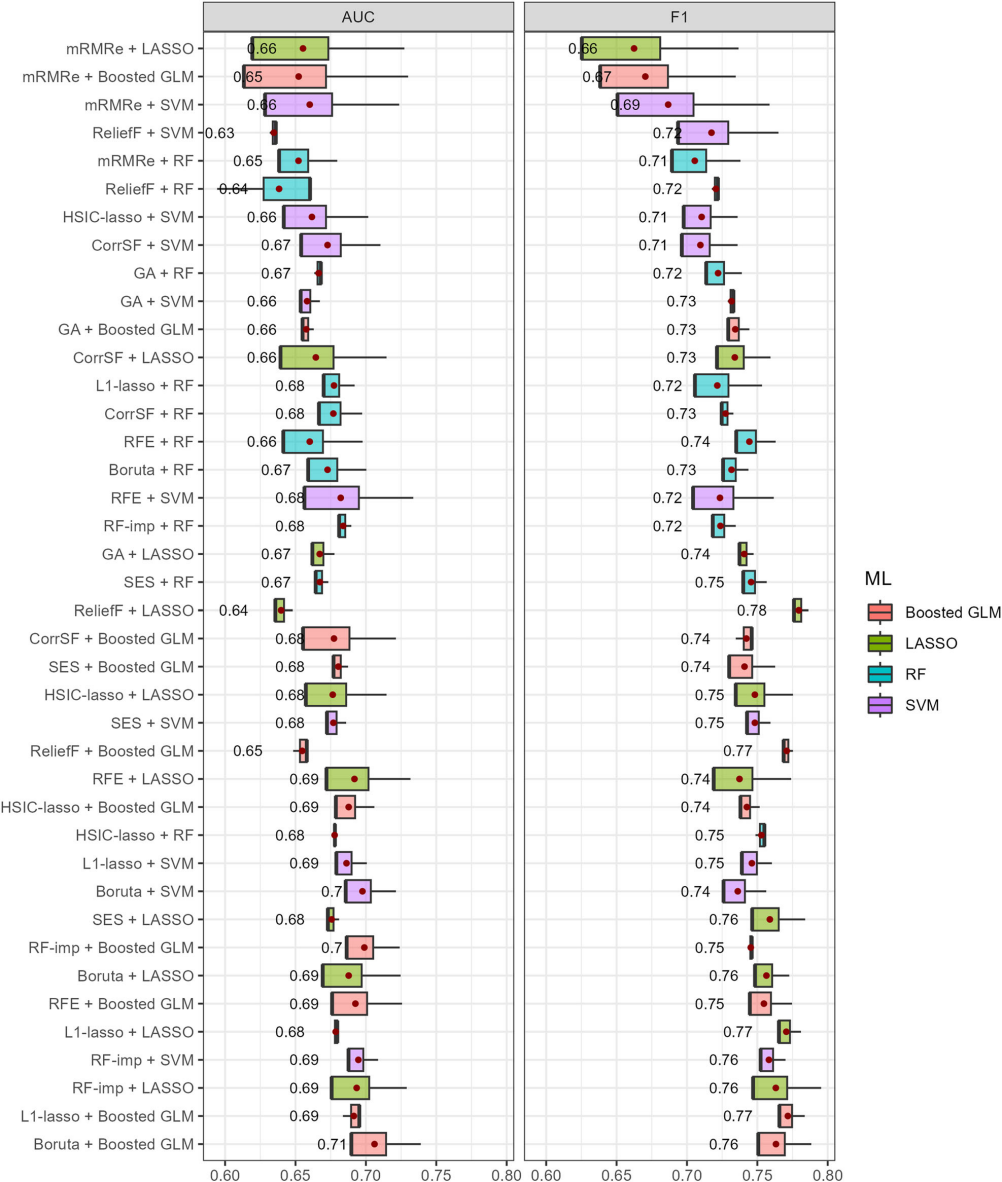
Boxplots of the AUC and F1 score for all the combinations of feature selection methods and ML classifiers in setting 1. The average performance (red points) is provided on the right side of the boxes

**Fig. 3:**
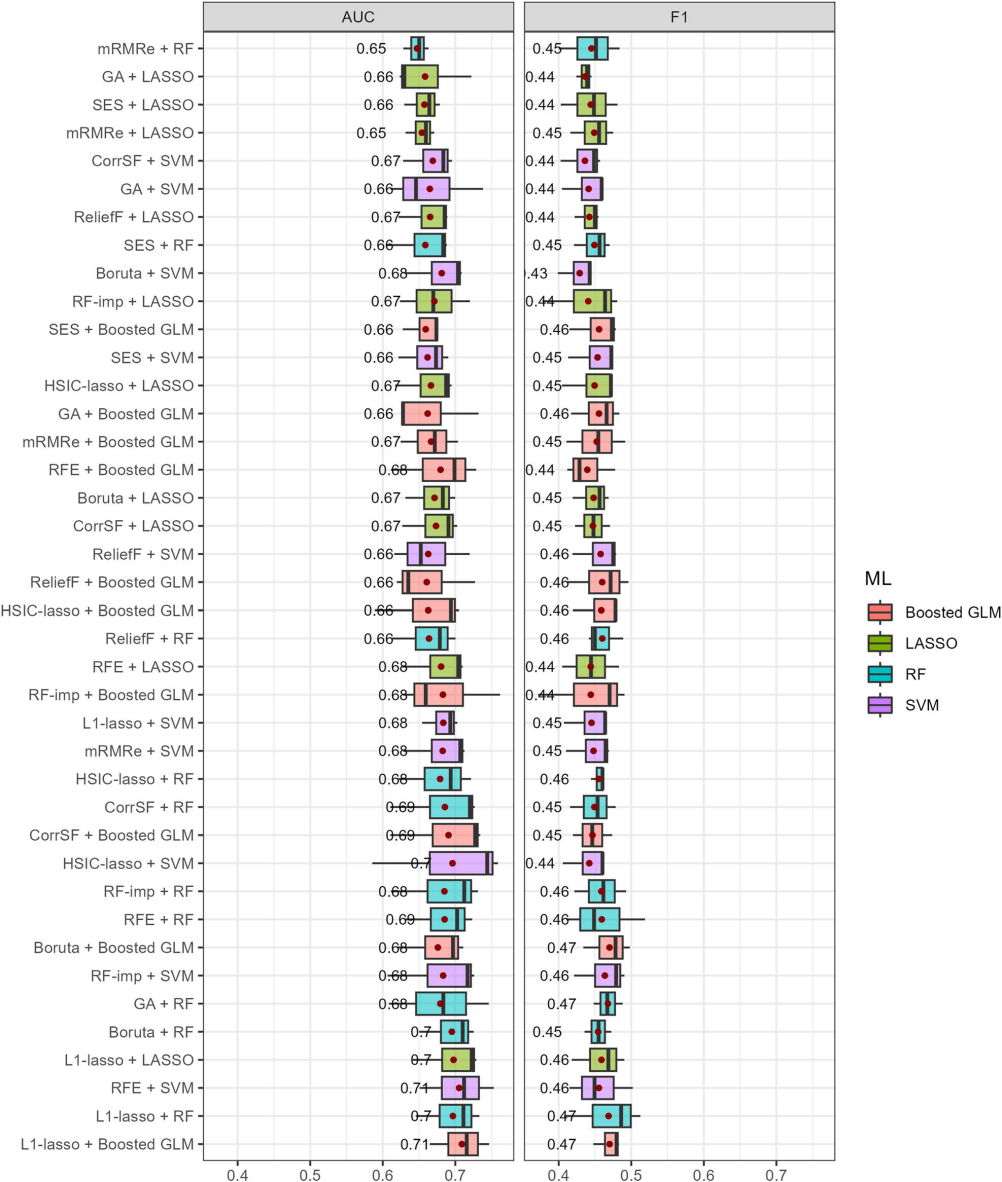
Boxplots of the AUC and F1 score for all the combinations of feature selection methods and ML classifiers in setting 2. The average performance (red points) is provided on the right side of the boxes

Considering the potential interactions between feature selection methods and ML classifiers, we evaluated whether certain combinations work better than others. The boxplots in Figs. 2 and 3 illustrate the mean performance for each combination of feature selection method and ML classifier, in settings 1 and 2, respectively. In setting 1, the best model resulted from the combination of Boruta with Boosted GLM (AUC = 0.71, F1 = 0.76). Boruta, RFE, L1-lasso, and RF-imp were among the top-performing feature selection methods, usually in combination with Boosted GLM and LASSO classifiers. Notably, models utilizing mRMRe had the highest variability. The best model in setting 2 resulted from the combination of L1-lasso with Boosted GLM (AUC = 0.71, F1 = 0.47). L1-lasso showed the overall best performance regardless of the choice of the classifier, while RFE, Boruta, and RF-imp were among the top-performing feature selection methods.

**Fig. 4:**
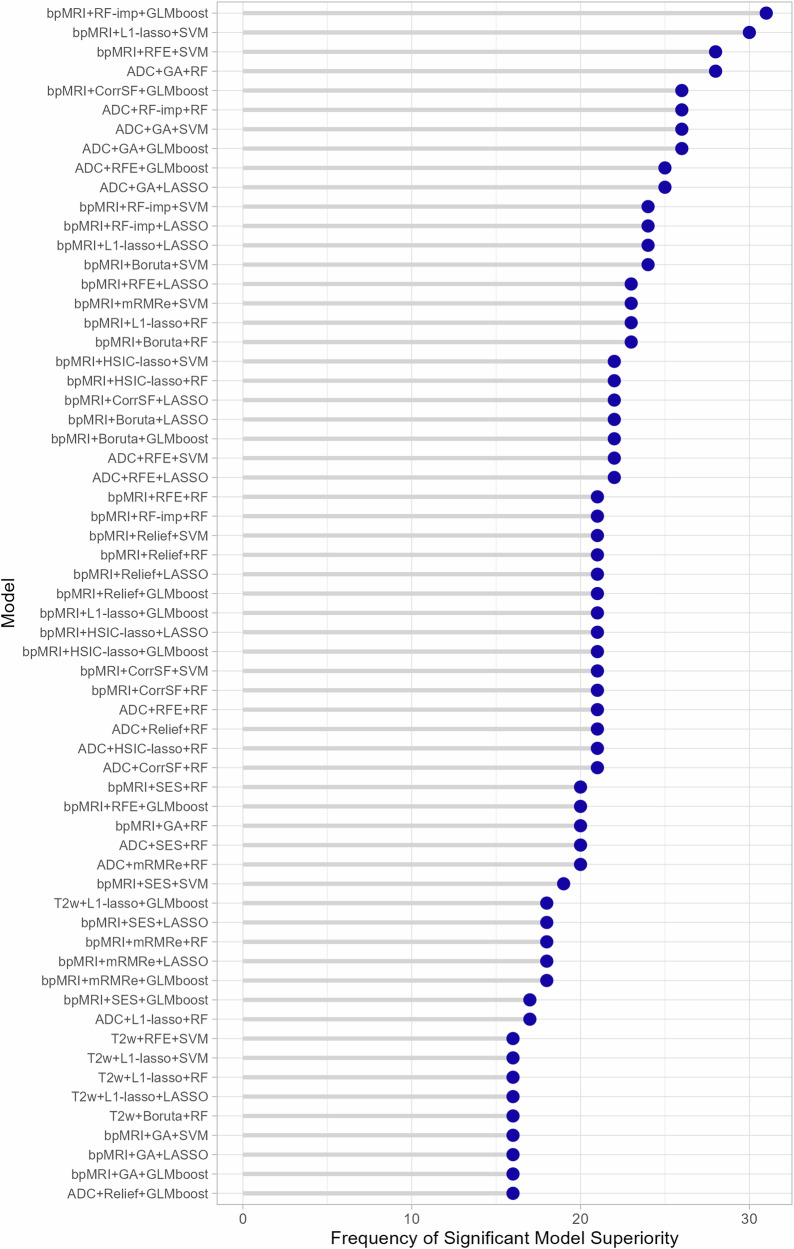
Models exhibiting statistically significant differences in ROC AUC in setting 2 and the frequency at which they outperformed other models in Delong’s test

Models that exhibited significantly superior performance in setting 2, as determined by Delong’s test, after correcting for multiple comparisons (*p*-adjusted = 0. 00042), are shown in Fig. 4. The *x*-axis indicates the frequency of a specific model being significantly better than others, while the models are listed on the *y*-axis in descending order of occurrence. Out of the 120 models trained and tested in the external validation setting, 62 exhibited at least once a significant superiority over other models. The most frequent statistically significant differences arose from the combination of bpMRI features with either RF-imp and Boosted GLM classifier, totaling 31 instances, or L1-lasso and SVM classifier, totaling 30 instances. The results of a grouping analysis, categorized based on MRI sequence, feature selection method, and ML classifier are presented in Supplementary Fig. S3.

### Comparison across ML classifiers and MRI sequences

The performance of the ML classifiers for settings 1 and 2, averaged across all the feature selection methods, is presented in Tables 4 and 5, respectively. The Boosted GLM exhibited a marginally improved performance in several cases, while a slight superiority of RF was only noticed in setting 2. Nevertheless, no discernible trend emerged to suggest the consistent superiority of any particular algorithm.

**Table 4:** Average performance (across feature selection methods and folds) for each ML classifier in setting 1

	ML algorithm	AUC		Balanced accuracy	F1	NPV	PPV	Sensitivity	Specificity
T2w	GLMboost	0.67 ± 0.04		0.62 ± 0.05	0.74 ± 0.05	0.38 ± 0.06	0.81 ± 0.06	0.68 ± 0.05	0.68 ± 0.06
	LASSO	0.66 ± 0.04		0.61 ± 0.04	0.73 ± 0.05	0.37 ± 0.06	0.8 ± 0.05	0.68 ± 0.07	0.68 ± 0.07
	RBF-SVM	0.66 ± 0.06		0.61 ± 0.06	0.71 ± 0.05	0.37 ± 0.08	0.81 ± 0.06	0.64 ± 0.04	0.64 ± 0.06
	RF	0.66 ± 0.03		0.62 ± 0.03	0.72 ± 0.03	0.37 ± 0.07	0.82 ± 0.05	0.65 ± 0.03	0.65 ± 0.05
ADC	GLMboost	0.72 ± 0.04		0.67 ± 0.04	0.76 ± 0.05	0.42 ± 0.06	0.84 ± 0.05	0.69 ± 0.07	0.64 ± 0.07
	LASSO	0.71 ± 0.03		0.66 ± 0.04	0.77 ± 0.04	0.42 ± 0.05	0.84 ± 0.05	0.71 ± 0.05	0.62 ± 0.06
	RBF-SVM	0.72 ± 0.05		0.68 ± 0.04	0.78 ± 0.03	0.43 ± 0.05	0.85 ± 0.05	0.71 ± 0.03	0.64 ± 0.06
	RF	0.71 ± 0.03		0.66 ± 0.03	0.75 ± 0.03	0.41 ± 0.06	0.84 ± 0.06	0.68 ± 0.03	0.64 ± 0.06
bpMRI	GLMboost	0.7 ± 0.04		0.66 ± 0.05	0.76 ± 0.04	0.41 ± 0.05	0.84 ± 0.06	0.7 ± 0.05	0.7 ± 0.05
	LASSO	0.7 ± 0.04		0.65 ± 0.04	0.77 ± 0.03	0.42 ± 0.07	0.83 ± 0.05	0.72 ± 0.06	0.72 ± 0.06
	RBF-SVM	0.7 ± 0.04		0.65 ± 0.04	0.75 ± 0.03	0.4 ± 0.07	0.83 ± 0.05	0.69 ± 0.05	0.69 ± 0.05
	RF	0.68 ± 0.04		0.64 ± 0.03	0.74 ± 0.03	0.39 ± 0.06	0.83 ± 0.04	0.67 ± 0.04	0.67 ± 0.04

**Table 5:** Average performance (across feature selection methods) for each ML classifier in setting 2

	ML algorithm	AUC	Balanced accuracy	F1	NPV	PPV	Sensitivity	Specificity
T2w	GLMboost	0.62 ± 0.02	0.57 ± 0.01	0.42 ± 0.02	0.81 ± 0.02	0.29 ± 0.01	0.7 ± 0.06	0.39 ± 0.08
	LASSO	0.63 ± 0.01	0.57 ± 0.01	0.42 ± 0.02	0.81 ± 0.02	0.29 ± 0.01	0.71 ± 0.08	0.39 ± 0.1
	RBF-SVM	0.62 ± 0.02	0.55 ± 0.02	0.41 ± 0.01	0.8 ± 0.01	0.29 ± 0.02	0.72 ± 0.05	0.36 ± 0.08
	RF	0.62 ± 0.02	0.59 ± 0.02	0.43 ± 0.02	0.81 ± 0.01	0.32 ± 0.01	0.67 ± 0.07	0.51 ± 0.05
ADC	GLMboost	0.7 ± 0.04	0.64 ± 0.01	0.48 ± 0.01	0.87 ± 0.01	0.34 ± 0.01	0.8 ± 0.04	0.48 ± 0.05
	LASSO	0.69 ± 0.02	0.62 ± 0.01	0.46 ± 0.02	0.87 ± 0.02	0.33 ± 0.01	0.8 ± 0.05	0.43 ± 0.06
	RBF-SVM	0.7 ± 0.03	0.62 ± 0.02	0.47 ± 0.02	0.87 ± 0.02	0.33 ± 0.01	0.79 ± 0.04	0.44 ± 0.03
	RF	0.71 ± 0.03	0.64 ± 0.02	0.48 ± 0.02	0.86 ± 0.01	0.35 ± 0.03	0.75 ± 0.04	0.47 ± 0.04
bpMRI	GLMboost	0.7 ± 0.04	0.63 ± 0.02	0.47 ± 0.03	0.87 ± 0.04	0.33 ± 0.02	0.81 ± 0.08	0.45 ± 0.04
	LASSO	0.69 ± 0.03	0.61 ± 0.02	0.46 ± 0.02	0.87 ± 0.03	0.32 ± 0.02	0.81 ± 0.07	0.4 ± 0.03
	RBF-SVM	0.71 ± 0.03	0.62 ± 0.02	0.46 ± 0.02	0.86 ± 0.02	0.32 ± 0.02	0.79 ± 0.05	0.43 ± 0.05
	RF	0.7 ± 0.02	0.63 ± 0.02	0.47 ± 0.03	0.85 ± 0.02	0.34 ± 0.03	0.74 ± 0.07	0.51 ± 0.09

### Performance variation explained

**Fig. 5:**
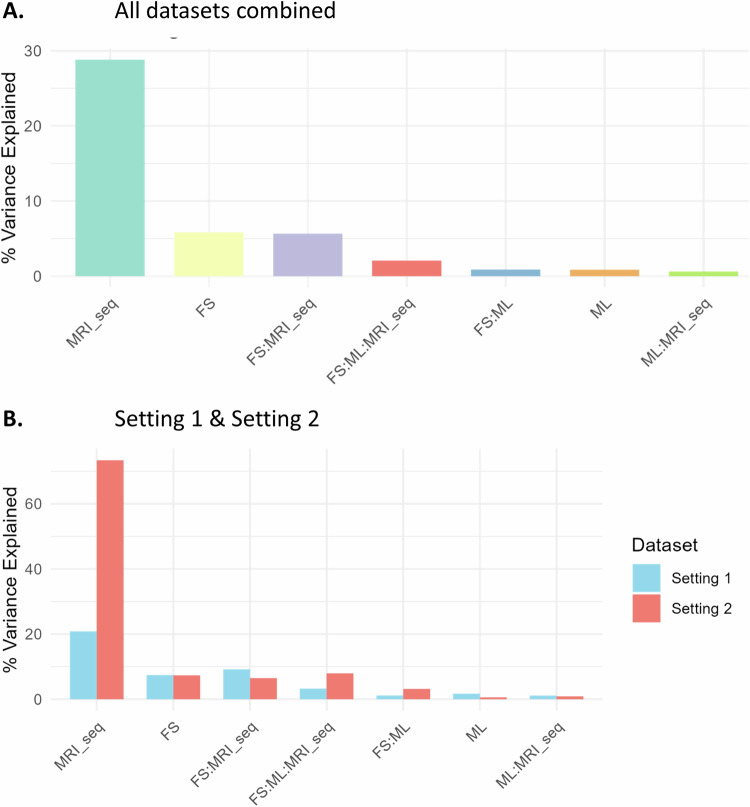
Variation of AUC explained by feature selection method, ML classifiers, and MRI sequence, and their interactions (**A**) for all settings, and (**B**) for settings 1 and 2, separately

Figure 5 shows what percentage of the AUC variation can be explained by different factors for all the experiments combined (Fig. 5A), and for the two settings separately (Fig. 5B). In total, the feature selection method, the classifier, the MRI sequence, and their interactions accounted for 45% of the variation in AUC. The MRI sequence was the most dominant source of variability as it explained 28.8% of the total variance in AUC scores (*F* = 4.17, *p* < 0.05). Feature selection accounted for 5.8% (*F* = 92.83, *p* < 0.05) and the interaction of feature selection and MRI sequence explained another 5.7% of the total variation (*F* = 2.02, *p* < 0.05). The effects of these factors/interactions on AUC were all statistically significant. Contrarily, the classifier and its interactions with other factors accounted for less than 2% of the total variance each, and the corresponding effects were non-significant. Notably, in setting 2, the MRI sequence explained 73% of the variation in AUC compared to 21% for setting 1.

### Radiomic features

**Fig. 6:**
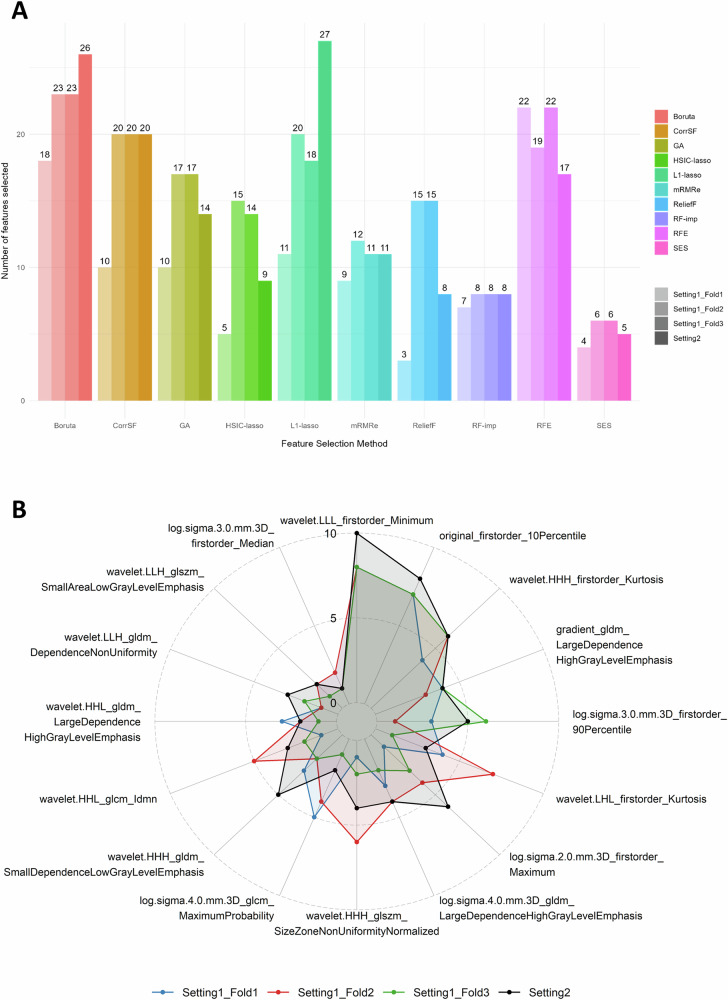
Comparative analysis of feature selection methods for ADC radiomic features across settings and folds. (**A**) Barplot depicting the number of selected features for each method, and (**B**) Radar plot illustrating the selection frequency for each feature aggregated across the ten feature selection methods

Considering the ADC-only radiomic feature set, Fig. 6A shows the number of features selected with each feature selection method and setting/fold. The number of features ranged from 3 to 27, with the Boruta (18–26 features) and RFE (17–22 features) selecting more features, while SES (4–6 features) and RF-imp (7–8 features) resulted in smaller feature subsets. The higher variability across settings/folds was observed for the L1-lasso (11–27 features).

Figure 6B shows the ADC radiomic features that were selected simultaneously in all settings/folds, along with details on their selection frequency across methods. Of the 16 features that were selected in all the experiments with at least one method, there was a good agreement regarding the frequency of selection for the four most frequently selected. Notably, the “*wavelet.LLL.firstorder_Minimum”* was consistently chosen across all methods in setting 2 and in 8 out of 10 methods in each fold of setting 1.

## Discussion

This study offers a comprehensive assessment of the impact of some of the most popular feature selection methods and ML classification algorithms on different combinations of bpMRI sequences, with a focus on PCa diagnosis. The effect of feature selection methods on models’ performance varied across experiments, while different ML algorithms resulted in similar performance scores. Therefore, the need for an informed approach to feature selection emerges as a critical determinant in optimizing the overall predictive performance in the context of radiomic-based PCa analyses. RFE, L1-lasso, RF-imp, and Boruta, demonstrated a consistent superiority for the majority of metrics. Similarly, in a recent study, RFE was highlighted as the most effective feature selection method for predicting csPCa from bpMRI [16].

Filter methods, such as mutual information-based methods, apply statistical approaches to remove features, usually on the basis of correlation or variance, without applying any predictive modeling. This is a popular choice for feature selection in radiomics studies due to its simplicity and efficiency [43]. Wrapper methods, like Boruta [44] and RFE [45], create a subset of features and apply this subset to a predictive model in order to evaluate its performance. These methods offer the advantage of taking into consideration correlations and interactions among radiomic features, partly overcoming the limitations of univariate filter methods, which only investigate the statistical relationship between the radiomics features. The main limitation of wrapper techniques is the high computational cost of finding the optimal set from a high-dimensional space, as well as the increased risk of overfitting. Embedded methods combine the advantages of filter and wrapper methods by performing feature selection during the generation of the ML model [46]. Examples of embedded feature selection methods include tree-based algorithms such as the RF classifier and the LASSO. The choice of feature selection methods implemented and evaluated in this work was guided by the need to encompass a comprehensive methodological spectrum [47]. A variety of filter, wrapper, and embedded methods were included with a particular focus on techniques adept at handling high-dimensional data, mitigating redundancy, and enhancing model interpretability.

For the classification task, we evaluated some of the most commonly utilized ML algorithms, ensuring methodological diversity while also considering previous evidence of their effectiveness in other radiomics-based comparative studies [48–50]. Boosted GLM showed a marginal superiority, but this trend lacked consistency. In general, ML classifiers resulted in similar performance scores across the experiments, suggesting that there is no algorithm that stands out as more robust than others. This aligns with existing literature, suggesting the choice of feature selection methods has a greater impact on prediction performance than the choice of the classifier [13, 16].

Undoubtedly, the source of radiomic features had the largest impact on the models’ performance. Combining multiple MRI sequences for feature extraction is common practice in PCa radiomics studies. Nevertheless, the added value of combining T2w with ADC or DWI features remains a topic of research [51]. Our study suggests that using solely ADC radiomic features leads to the most robust models, as the integration of T2w-derived features did not yield any significant improvements. Similarly, in a study comparing bpMRI sequences for distinguishing high-grade PCa, there was no significant difference in AUC between combined T2w-DWI models and DWI-only models [52]. In another study, adding T2w information to a PCa detection model even reduced predictive performance compared to the ADC-based model [53].

Certain limitations of our study should also be acknowledged. Specifically, the effect of eliminating redundant (within-feature correlation) and irrelevant (feature-outcome correlation) features, prior to the exhaustive feature selection, was not evaluated. Additionally, it is possible that incorporating clinical variables into radiomics models or combining multiple feature selection methods would lead to improved models’ performance.

While this work serves as a valuable resource for improving radiomics workflows in diagnosing csPCa, caution is advised in interpreting the results. Further research is needed to determine which feature selection method and ML algorithm are more robust, stable, and versatile for radiomics applications. Their efficacy should be confirmed on different MRI datasets, not only for the diagnosis of csPCa, but ideally for different clinical scenarios. Additionally, future research efforts should prioritize the development and evaluation of robust feature selection strategies to further advance the reliability and generalizability of radiomics models. Particularly, deep learning-based feature selection, such as unsupervised techniques based on autoencoders [54, 55], is an emerging topic in radiomics research that could potentially improve classification results compared to conventional methods [56].

Our findings may guide future radiomic research paving the way for the development of more effective and reliable radiomic models not only for advancing PCa diagnosis strategies, but also for informing broader applications of radiomics in different medical contexts.

## Supplementary information

ELECTRONIC SUPPLEMENTARY MATERIAL

## Data Availability

The data underlying this article will be shared on reasonable request to the corresponding author.

## Abbreviations

ADC: Apparent diffusion coefficient
AI: Artificial intelligence
AUC: Area under the receiver operating characteristic curve
bpMRI: Biparametric MRI
CLEAR: CheckList for EvaluAtion of radiomics research
CorrSF: Correlation-based feature selection with forward selection strategy
csPCa: Clinically significant prostate cancer
CV: Cross-validation
GA: Genetic algorithm
GLM: Generalized linear model
HSIC: Hilbert-Schmidt independence criterion
LASSO: Least absolute shrinkage and selection operator
METRICS: METhodological RadiomICs score
mpMRI: Multiparametric MRI
mRMRe: Minimum redundancy maximum relevance ensemble
NPV: Negative predictive value
PCa: Prostate cancer
PPV: Positive predictive value
RF: Random forest
RFE: Recursive feature elimination
RF-imp: Random forest variable importance
SES: Statistically equivalent multiple feature subsets
SVM: Support vector machine

## References

[1] Rebello RJ, Oing C, Knudsen KE et al (2021) Prostate cancer. Nat Rev Dis Prim 7:1–27 33542230 10.1038/s41572-020-00243-0

[2] Ahmed HU, El-Shater Bosaily A, Brown LC et al (2017) Diagnostic accuracy of multi-parametric MRI and TRUS biopsy in prostate cancer (PROMIS): a paired validating confirmatory study. Lancet 389:815–822 28110982 10.1016/S0140-6736(16)32401-1

[3] Goldenberg SL, Nir G, Salcudean SE (2019) A new era: artificial intelligence and machine learning in prostate cancer. Nat Rev Urol 16:391–403 31092914 10.1038/s41585-019-0193-3

[4] Guiot J, Vaidyanathan A, Deprez L et al (2022) A review in radiomics: making personalized medicine a reality via routine imaging. Med Res Rev 42:426–440 34309893 10.1002/med.21846

[5] Lambin P, Leijenaar RTH, Deist TM et al (2017) Radiomics: the bridge between medical imaging and personalized medicine. Nat Rev Clin Oncol 14:749–762 28975929 10.1038/nrclinonc.2017.141

[6] Hunter B, Hindocha S, Lee RW (2022) The role of artificial intelligence in early cancer diagnosis. Cancers (Basel) 14:1524 35326674 10.3390/cancers14061524 PMC8946688

[7] Koh D-M, Papanikolaou N, Bick U et al (2022) Artificial intelligence and machine learning in cancer imaging. Commun Med 2:1–14 36310650 10.1038/s43856-022-00199-0 PMC9613681

[8] van Timmeren JE, Cester D, Tanadini-Lang S, Alkadhi H, Baessler B (2020) Radiomics in medical imaging—“how-to” guide and critical reflection. Insights Imaging 11:1–16 32785796 10.1186/s13244-020-00887-2 PMC7423816

[9] Demircioğlu A (2022) Evaluation of the dependence of radiomic features on the machine learning model. Insights Imaging 13:1–11 35201534 10.1186/s13244-022-01170-2 PMC8873309

[10] Ibrahim A, Primakov S, Beuque M et al (2021) Radiomics for precision medicine: current challenges, future prospects, and the proposal of a new framework. Methods 188:20–29 32504782 10.1016/j.ymeth.2020.05.022

[11] Zhang YP, Zhang XY, Cheng YT et al (2023) Artificial intelligence-driven radiomics study in cancer: the role of feature engineering and modeling. Mil Med Res 10:1–33 37189155 10.1186/s40779-023-00458-8 PMC10186733

[12] Zhang W, Guo Y, Jin Q, Zhang W, Guo Y, Jin Q (2023) Radiomics and its feature selection: a review. Symmetry 15:1834

[13] Demircioǧlu A (2022) Benchmarking feature selection methods in radiomics. Invest Radiol 57:433–443 35045555 10.1097/RLI.0000000000000855

[14] Parmar C, Grossmann P, Bussink J, Lambin P, Aerts HJWL (2015) Machine learning methods for quantitative radiomic biomarkers. Sci Rep 5:1–11 10.1038/srep13087 PMC4538374 26278466

[15] Decoux A, Duron L, Habert P et al (2023) Comparative performances of machine learning algorithms in radiomics and impacting factors. Sci Rep 13:1–10 37640728 10.1038/s41598-023-39738-7 PMC10462640

[16] Rodrigues A, Santinha J, Galvão B, Matos C, Couto FM, Papanikolaou N (2021) Prediction of prostate cancer disease aggressiveness using bi-parametric MRI radiomics. Cancers (Basel) 13:6065 34885175 10.3390/cancers13236065 PMC8657292

[17] Kumar A, Jha AK, Agarwal JP et al (2023) Machine-learning-based radiomics for classifying glioma grade from magnetic resonance images of the brain. J Pers Med 13:920 37373909 10.3390/jpm13060920 PMC10305272

[18] Corso F, Tini G, Lo Presti G et al (2021) The challenge of choosing the best classification method in radiomic analyses: recommendations and applications to lung cancer CT images. Cancers (Basel) 13:3088 34205631 10.3390/cancers13123088 PMC8234634

[19] Chen C, Zheng A, Ou X, Wang J, Ma X (2020) Comparison of radiomics-based machine-learning classifiers in diagnosis of glioblastoma from primary central nervous system lymphoma. Front Oncol 10:1151 33042784 10.3389/fonc.2020.01151 PMC7522159

[20] Destito M, Marzullo A, Leone R et al (2022) Radiomics-based machine learning model for predicting overall and progression-free survival in rare cancer: a case study for primary CNS lymphoma patients. Bioengineering 10:285 10.3390/bioengineering10030285 PMC10045100 36978676

[21] Schwier M, van Griethuysen J, Vangel MG et al (2019) Repeatability of multiparametric prostate MRI radiomics features. Sci Rep 9:1–16 31263116 10.1038/s41598-019-45766-z PMC6602944

[22] Donisi L, Cesarelli G, Castaldo A et al (2021) A combined radiomics and machine learning approach to distinguish clinically significant prostate lesions on a publicly available MRI dataset. J Imaging 7:215 34677301 10.3390/jimaging7100215 PMC8540196

[23] Isaksson LJ, Raimondi S, Botta F et al (2020) Effects of MRI image normalization techniques in prostate cancer radiomics. Phys Medica 71:7–13 10.1016/j.ejmp.2020.02.007 32086149

[24] Bernatz S, Ackermann J, Mandel P et al (2020) Comparison of machine learning algorithms to predict clinically significant prostate cancer of the peripheral zone with multiparametric MRI using clinical assessment categories and radiomic features. Eur Radiol 30:6757–6769 32676784 10.1007/s00330-020-07064-5 PMC7599168

[25] Bleker J, Kwee TC, Dierckx RAJO, de Jong IJ, Huisman H, Yakar D (2020) Multiparametric MRI and auto-fixed volume of interest-based radiomics signature for clinically significant peripheral zone prostate cancer. Eur Radiol 30:1313–1324 31776744 10.1007/s00330-019-06488-y PMC7033141

[26] Chen T, Zhang Z, Tan S et al (2022) MRI based radiomics compared with the PI-RADS V2.1 in the prediction of clinically significant prostate cancer: biparametric vs multiparametric MRI. Front Oncol 11:792456 35127499 10.3389/fonc.2021.792456 PMC8810653

[27] ProCAncer-I (2023) An AI platform integrating imaging data and models, supporting precision care through prostate cancer’s continuum. https://www.procancer-i.eu/. Accessed 13 Nov 2023

[28] Samuel G, Armato I, Huisman H et al (2018) PROSTATEx challenges for computerized classification of prostate lesions from multiparametric magnetic resonance images. J Med Imaging 5:1 10.1117/1.JMI.5.4.044501 PMC6228312 30840739

[29] Cuocolo R, Stanzione A, Castaldo A, De Lucia DR, Imbriaco M (2021) Quality control and whole-gland, zonal and lesion annotations for the PROSTATEx challenge public dataset. Eur J Radiol 138:109647 33721767 10.1016/j.ejrad.2021.109647

[30] Cuocolo R, Comelli A, Stefano A et al (2021) Deep learning whole-gland and zonal prostate segmentation on a public MRI dataset. J Magn Reson Imaging 54:452–459 33634932 10.1002/jmri.27585

[31] Mottet N, Bellmunt J, Bolla M et al (2017) EAU-ESTRO-SIOG guidelines on prostate cancer. Part 1: screening, diagnosis, and local treatment with curative intent. Eur Urol 71:618–629 27568654 10.1016/j.eururo.2016.08.003

[32] Briganti A, Fossati N, Catto JWF et al (2018) Active surveillance for low-risk prostate cancer: the European Association of Urology Position in 2018. Eur Urol 74:357–368 29937198 10.1016/j.eururo.2018.06.008

[33] Tustison NJ, Avants BB, Cook PA et al (2010) N4ITK: improved N3 bias correction. IEEE Trans Med Imaging 29:1310–1320 20378467 10.1109/TMI.2010.2046908 PMC3071855

[34] Van Griethuysen JJM, Fedorov A, Parmar C et al (2017) Computational radiomics system to decode the radiographic phenotype. Cancer Res 77:e104–e107 29092951 10.1158/0008-5472.CAN-17-0339 PMC5672828

[35] Duron L, Balvay D, Vande Perre S et al (2019) Gray-level discretization impacts reproducible MRI radiomics texture features. PLoS One 14:e0213459 10.1371/journal.pone.0213459 PMC6405136 30845221

[36] Tixier F, Le Rest CC, Hatt M et al (2011) Intratumor heterogeneity characterized by textural features on baseline 18F-FDG PET images predicts response to concomitant radiochemotherapy in esophageal cancer. J Nucl Med 52:369–378 21321270 10.2967/jnumed.110.082404 PMC3789272

[37] Haynes W (2013) Benjamini–Hochberg method. In: Encyclopedia of systems biology. Springer, Berlin, p 78

[38] Tibshirani R (1996) Regression shrinkage and selection via the lasso. J R Stat Soc Ser B 58:267–288

[39] Gretton A, Bousquet O, Smola A, Scḧlkopf B (2005) Measuring statistical dependence with Hilbert-Schmidt norms. In: Lecture notes in computer science (including subseries lecture notes in artificial intelligence and lecture notes in bioinformatics), vol 3734 LNAI, pp 63–77

[40] Park JE, Park SY, Kim HJ, Kim HS (2019) Reproducibility and generalizability in radiomics modeling: possible strategies in radiologic and statistical perspectives. Korean J Radiol 20:1124–1137 31270976 10.3348/kjr.2018.0070 PMC6609433

[41] Kocak B, Baessler B, Bakas S et al (2023) CheckList for EvaluAtion of Radiomics research (CLEAR): a step-by-step reporting guideline for authors and reviewers endorsed by ESR and EuSoMII. Insights Imaging 14:1–13 37142815 10.1186/s13244-023-01415-8 PMC10160267

[42] Kocak B, Akinci D’Antonoli T, Mercaldo N et al (2024) METhodological RadiomICs Score (METRICS): a quality scoring tool for radiomics research endorsed by EuSoMII. Insights Imaging 15:1–18 38228979 10.1186/s13244-023-01572-w PMC10792137

[43] Bommert A, Sun X, Bischl B, Rahnenführer J, Lang M (2020) Benchmark for filter methods for feature selection in high-dimensional classification data. Comput Stat Data Anal 143:106839

[44] Kursa MB, Rudnicki WR (2010) Feature selection with the boruta package. J Stat Softw 36:1–13

[45] Darst BF, Malecki KC, Engelman CD (2018) Using recursive feature elimination in random forest to account for correlated variables in high dimensional data. BMC Genet 19:1–6 30255764 10.1186/s12863-018-0633-8 PMC6157185

[46] Dinov ID (2018) Variable/feature selection. In Data Science and Predictive Analytics: Biomedical and Health Applications using R, Springer, Cham, pp 557–572

[47] Pudjihartono N, Fadason T, Kempa-Liehr AW, O’Sullivan JM (2022) A review of feature selection methods for machine learning-based disease risk prediction. Front Bioinform 2:927312 36304293 10.3389/fbinf.2022.927312 PMC9580915

[48] Yin P, Mao N, Zhao C et al (2019) Comparison of radiomics machine-learning classifiers and feature selection for differentiation of sacral chordoma and sacral giant cell tumour based on 3D computed tomography features. Eur Radiol 29:1841–1847 30280245 10.1007/s00330-018-5730-6

[49] Van Gómez O, Herraiz JL, Udías JM et al (2022) Analysis of cross-combinations of feature selection and machine-learning classification methods based on [18F]F-FDG PET/CT radiomic features for metabolic response prediction of metastatic breast cancer lesions. Cancers (Basel) 14:2922 35740588 10.3390/cancers14122922 PMC9221062

[50] Qian Z, Zhang L, Hu J et al (2021) Machine learning-based analysis of magnetic resonance radiomics for the classification of gliosarcoma and glioblastoma. Front Oncol 11:699789 34490097 10.3389/fonc.2021.699789 PMC8417735

[51] Huynh LM, Hwang Y, Taylor O, Baine MJ (2023) The use of MRI-derived radiomic models in prostate cancer risk stratification: a critical review of contemporary literature. Diagnostics 13:1128 36980436 10.3390/diagnostics13061128 PMC10047271

[52] Gong L, Xu M, Fang M et al (2020) Noninvasive prediction of high-grade prostate cancer via biparametric MRI radiomics. J Magn Reson Imaging 52:1102–1109 32212356 10.1002/jmri.27132

[53] Li C, Deng M, Zhong X et al (2023) Multi-view radiomics and deep learning modeling for prostate cancer detection based on multi-parametric MRI. Front Oncol 13:1198899 37448515 10.3389/fonc.2023.1198899 PMC10338012

[54] Hassanpour R, Netten N, Busker T, Shoae Bargh M, Choenni S (2023) Adaptive feature selection using an autoencoder and classifier: applied to a radiomics case. Proceedings of the 38th ACM/SIGAPP symposium on applied computing. SIGAPP, pp 1256–1259

[55] Sharifipour S, Fayyazi H, Sabokrou M, Adeli E (2019) Unsupervised feature ranking and selection based on autoencoders. ICASSP, IEEE International Conference on Acoustics, Speech, and Signal Processing. IEEE, Brighton, pp 3172–3176

[56] Haueise T, Liebgott A, Yang B (2022) A comparative study on the potential of unsupervised deep learning-based feature selection in radiomics. Annu Int Conf IEEE Eng Med Biol Soc 2022:541–544 10.1109/EMBC48229.2022.9871257 36085959

