# Supplementary material for: Optimizing radiomics for prostate cancer diagnosis: feature selection strategies, machine learning classifiers, and MRI sequences
Source: Insights Imaging. 2024 Nov 4;15:265. doi: 10.1186/s13244-024-01783-9 (PMC11535140; doi:10.1186/s13244-024-01783-9)

Optimizing Radiomics for Prostate Cancer Diagnosis: Feature Selection Strategies, Machine learning Classifiers, and MRI sequences

ELECTRONIC SUPPLEMENTARY MATERIAL

Supplementary Table S1. Configuration of PyRadiomics parameters used for radiomic feature extraction.

Pyradiomics Parameters	Parameters Values
**"imageType"**	• Original,<br>• Wavelet,<br>• Gradient,<br>• LoG with sigma: 2mm, 3mm, 4mm and 5mm.
"**featureClass**"	• firstorder<br>• glcm<br>• glrlm<br>• glszm<br>• gldm<br>• shape
"**settings**"	resampledPixelSpacing: [1, 1, 1]<br>normalize: True<br>normalizeScale: 100<br>padDistance: 5<br>binWidth:<br>• ProstateNET:<br>○ T2: 7<br>○ ADC: 7<br>• ProstateX2:<br>○ T2: 6<br>○ ADC: 3
Parameters other than those reported were set to default.	

Supplementary Table S2. CheckList for EvaluAtion of Radiomics research (CLEAR checklist)

Section	No	Item	Yes	No	n/a	Page
**Title**						
	1	Relevant title, specifying the radiomic methodology	☑	☐	☐	1
**Abstract**						
	2	Structured summary with relevant information	☑	☐	☐	1
**Keywords**						
	3	Relevant keywords for radiomics	☑	☐	☐	2
**Introduction**						
	4	Scientific or clinical background	☑	☐	☐	3
	5	Rationale for using a radiomic approach	☑	☐	☐	3-4
	6	Study objective(s)	☑	☐	☐	4
**Method**						
Study Design	7	Adherence to guidelines or checklists (e.g., CLEAR checklist)	☑	☐	☐	9
	8	Ethical details (e.g., approval, consent, data protection)	☑	☐	☐	Ethics declarations
	9	Sample size calculation	☐	☑	☐	-
	10	Study nature (e.g., retrospective, prospective)	☑	☐	☐	4
	11	Eligibility criteria	☑	☐	☐	4-5
	12	Flowchart for technical pipeline	☑	☐	☐	Fig. 1
Data	13	Data source (e.g., private, public)	☑	☐	☐	4
	14	Data overlap	☑	☐	☐	4-5
	15	Data split methodology	☑	☐	☐	8
	16	Imaging protocol (i.e., image acquisition and processing)	☑	☐	☐	5
	17	Definition of non-radiomic predictor variables	☐	☑	☐	Beyond the intended focus of this study
	18	Definition of the reference standard (i.e., outcome variable)	☑	☐	☐	5
Segmentation	19	Segmentation strategy	☑	☐	☐	4-5
	20	Details of operators performing segmentation	☐	☑	☐	Segmentations performed by experts across 12 clinical centers.
Pre-processing	21	Image pre-processing details	☑	☐	☐	5
	22	Resampling method and its parameters	☑	☐	☐	5
	23	Discretization method and its parameters	☑	☐	☐	5

	24	Image types (e.g., original, filtered, transformed)	☑	☐	☐	5-6
Feature extraction	25	Feature extraction method	☑	☐	☐	5
	26	Feature classes	☑	☐	☐	5-6
	27	Number of features	☑	☐	☐	6
	28	Default configuration statement for remaining parameters	☑	☐	☐	Suppl. Table 1
Data preparation	29	Handling of missing data	☐	☐	☑	No missing data
	30	Details of class imbalance	☑	☐	☐	Table 1
	31	Details of segmentation reliability analysis	☐	☑	☐	
	32	Feature scaling details (e.g., normalization, standardization)	☑	☐	☐	5
	33	Dimension reduction details	☑	☐	☐	6-8
Modeling	34	Algorithm details	☑	☐	☐	8
	35	Training and tuning details	☑	☐	☐	8
	36	Handling of confounders	☑	☐	☐	8
	37	Model selection strategy	☑	☐	☐	8
Evaluation	38	Testing technique (e.g., internal, external)	☑	☐	☐	8
	39	Performance metrics and rationale for choosing	☑	☐	☐	8-9
	40	Uncertainty evaluation and measures (e.g., confidence intervals)	☑	☐	☐	8
	41	Statistical performance comparison (e.g., DeLong's test)	☑	☐	☐	8
	42	Comparison with non-radiomic and combined methods	☐	☑	☐	Beyond the intended focus of this study
	43	Interpretability and explainability methods	☐	☑	☐	Beyond the intended focus of this study
**Results**						
	44	Baseline demographic and clinical characteristics	☑	☐	☐	Table 1
	45	Flowchart for eligibility criteria	☐	☐	☑	
	46	Feature statistics (e.g., reproducibility, feature selection)	☑	☐	☐	Fig.6
	47	Model performance evaluation	☑	☐	☐	Tables 2-5, Fig. 2-5
	48	Comparison with non-radiomic and combined approaches	☐	☑	☐	Beyond the intended focus of this study
**Discussion**						
	49	Overview of important findings	☑	☐	☐	12
	50	Previous works with differences from the current study	☑	☐	☐	13
	51	Practical implications	☑	☐	☐	14

	52	Strengths and limitations (e.g., bias and generalizability issues)	☑	☐	☐	14
**Open Science**						
Data availability	53	Sharing images along with segmentation data [n/e]	☐	☐	☑	Data privacy constraints
	54	Sharing radiomic feature data	☐	☐	☑	Data privacy constraints
Code availability	55	Sharing pre-processing scripts or settings	☑	☐	☐	Supp. Table 1
	56	Sharing source code for modeling	☐	☑	☐	Available upon request
Model availability	57	Sharing final model files	☐	☑	☐	Available upon request
	58	Sharing a ready-to-use system [n/e]	☐	☑	☐	

Yes, details provided; **No**, details not provided; **n/e**, not essential; **n/a**, not applicable

Supplementary Table S3. METhodological RadiomICs Score (METRICS)

Items/Conditions	Definitions	Weights	Answers
**Study Design**			
Item#1	Adherence to radiomics and/or machine learning-specific checklists or guidelines	0.0368	yes
Item#2	Eligibility criteria that describe a representative study population	0.0735	yes
Item#3	High-quality reference standard with a clear definition	0.0919	yes
**Imaging Data**			
Item#4	Multi-center	0.0438	yes
Item#5	Clinical translatability of the imaging data source for radiomics analysis	0.0292	yes
Item#6	Imaging protocol with acquisition parameters	0.0438	no
Item#7	The interval between imaging used and reference standard	0.0292	yes
**Segmentation**			
Condition#1	Does the study include segmentation?		yes
Condition#2	Does the study include fully automated segmentation?		no
Item#8	Transparent description of segmentation methodology	0.0337	no
Item#9	Formal evaluation of fully automated segmentation	0.0225	n/a
Item#10	Test set segmentation masks produced by a single reader or automated tool	0.0112	no
**Image Processing and Feature Extraction**			
Condition#3	Does the study include hand-crafted feature extraction?		yes
Item#11	Appropriate use of image preprocessing techniques with transparent description	0.0622	yes
Item#12	Use of standardized feature extraction software	0.0311	yes
Item#13	Transparent reporting of feature extraction parameters, otherwise providing a default configuration statement	0.0415	yes
**Feature Processing**			
Condition#4	Does the study include tabular data?		yes
Condition#5	Does the study include end-to-end deep learning?		no
Item#14	Removal of non-robust features	0.0200	yes
Item#15	Removal of redundant features	0.0200	yes
Item#16	Appropriateness of dimensionality compared to data size	0.0300	yes
Item#17	Robustness assessment of end-to-end deep learning pipelines	0.0200	n/a
**Preparation for Modeling**			
Item#18	Proper data partitioning process	0.0599	yes
Item#19	Handling of confounding factors	0.0300	yes
**Metrics and Comparison**			
Item#20	Use of appropriate performance evaluation metrics for task	0.0352	yes

Item#21	Consideration of uncertainty	0.0234	yes
Item#22	Calibration assessment	0.0176	no
Item#23	Use of uni-parametric imaging or proof of its inferiority	0.0117	yes
Item#24	Comparison with a non-radiomic approach or proof of added clinical value	0.0293	no
Item#25	Comparison with simple or classical statistical models	0.0176	no
**Testing**			
Item#26	Internal testing	0.0375	yes
Item#27	External testing	0.0749	yes
**Open Science**			
Item#28	Data availability	0.0075	no
Item#29	Code availability	0.0075	no
Item#30	Model availability	0.0075	no
**Total METRICS score: 81.7%**			
**Quality category *: Excellent**			
* 0≤score<20%, “very low”; 20≤score<40%, “low”; 40≤score<60%, “moderate”; 60≤score<80%, “good”; 80≤score≤100%, “excellent” quality			

Supplementary Figure S1. Average models' performance in Setting 1 (across folds) for each feature selection method, ML classifier and MRI sequence. Red boxes indicate to the best performing combination of feature selection methods and ML classifiers for each metric.

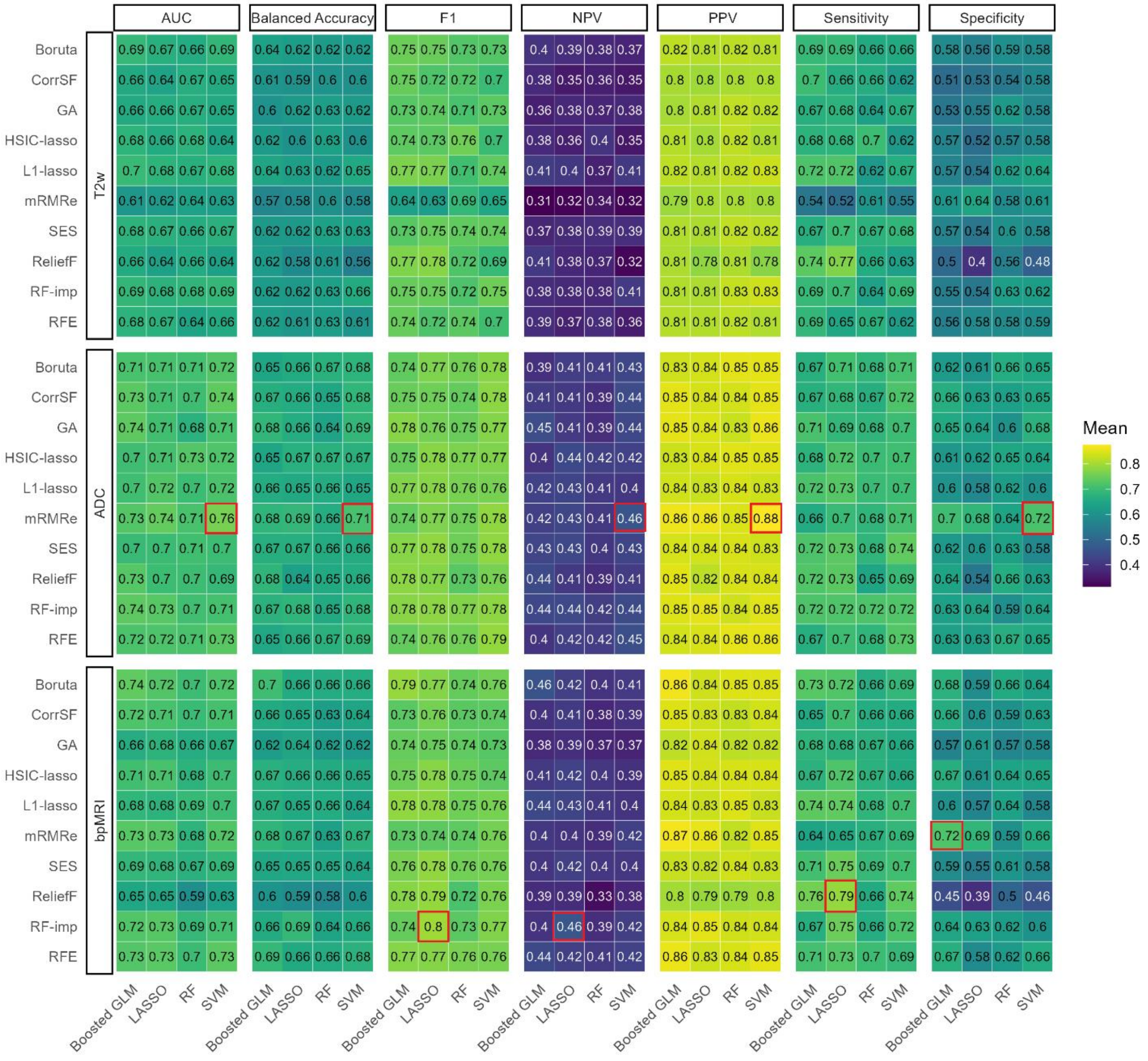

Supplementary Figure S2. Models' performance in Setting 2 for each feature selection method, ML classifier and MRI sequence. Red boxes indicate to the best performing combination of feature selection methods and ML classifiers for each metric.

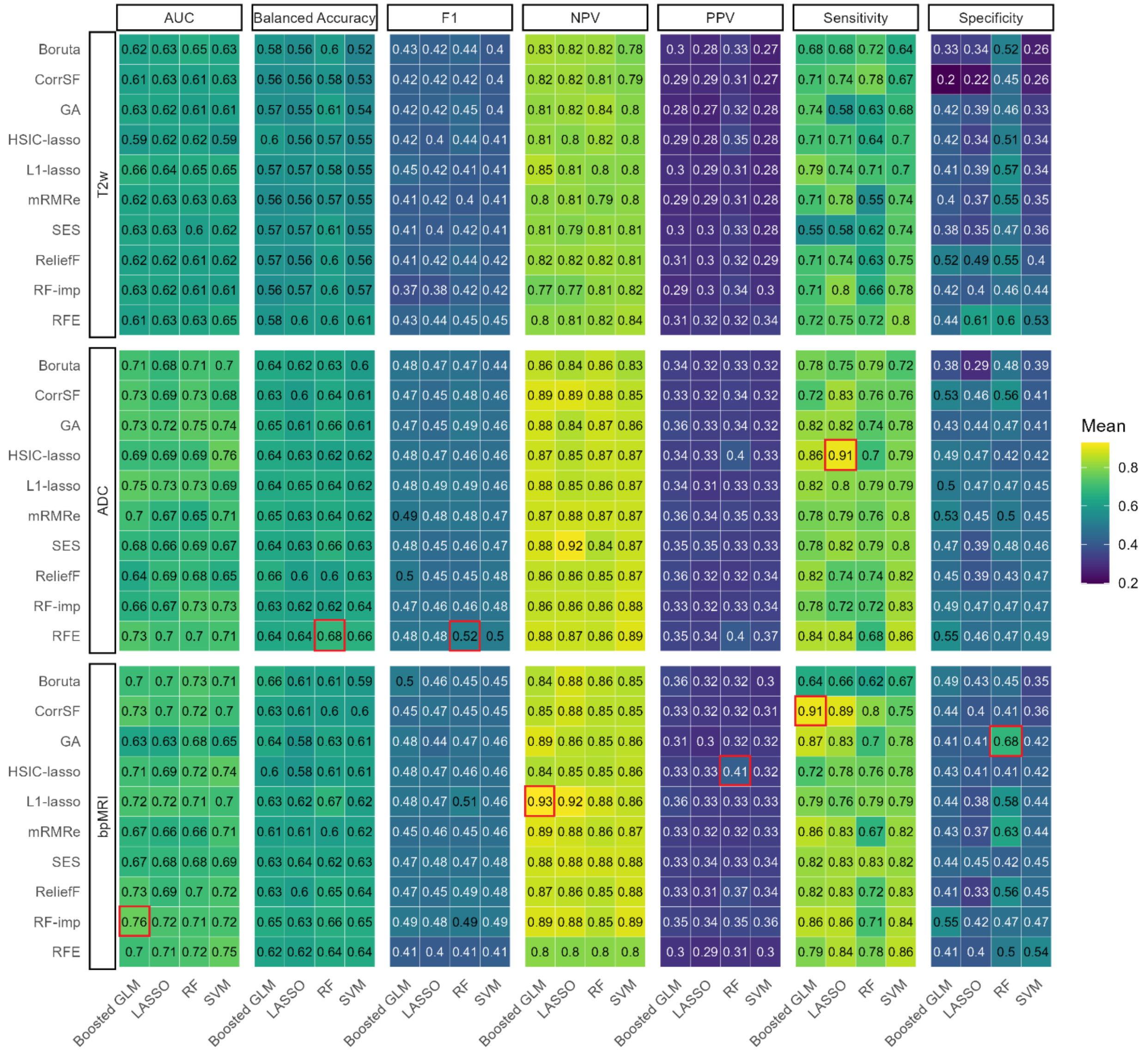

Supplementary Figure S3. Results of Delong's test grouped based on (A) the MRI sequence, (B) the Feature Selection method, and (C) the ML classifier. Each colored point indicates how many times a specific model was found to have significantly higher ROC AUC than others. The superiority of certain groups can be determined by the larger number of points (models) and the larger number of counts per model. Specifically, significant differences were found mostly for models containing either bpMRI or solely ADC features, combined with RF-imp, L1-lasso and RFE feature selection methods. Models trained with RF, SVM or Boosted GLM had a marginal superiority to the ones based on LASSO classifier.

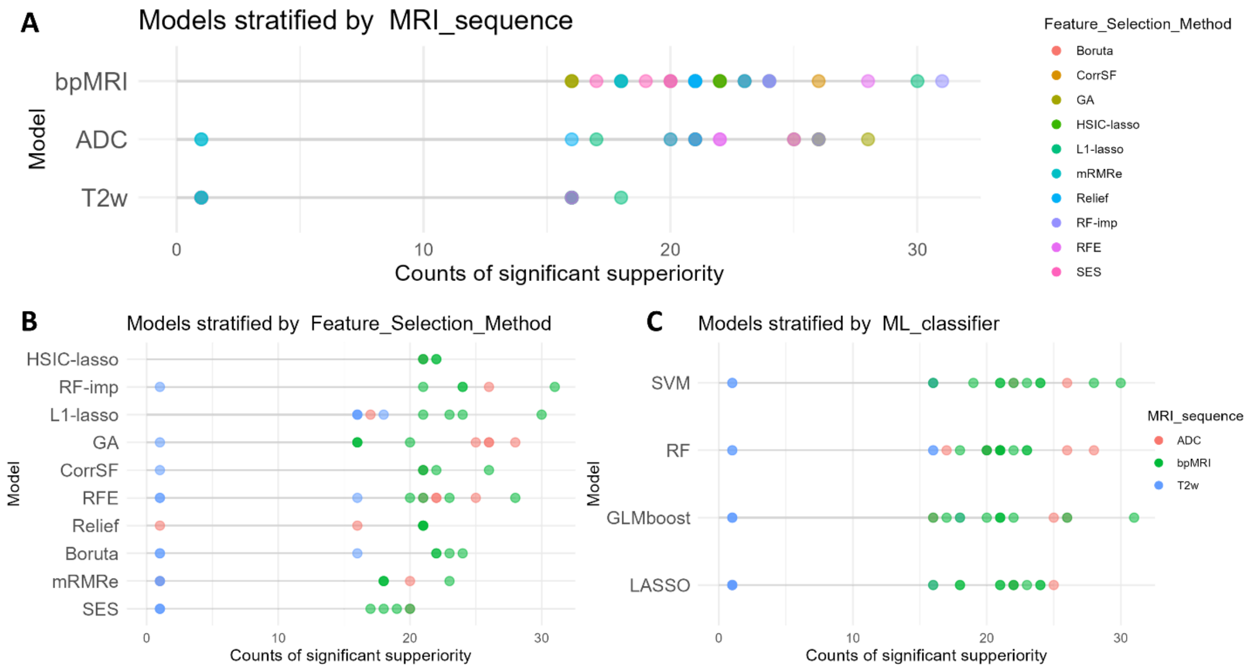

Supplement: Supplementary file 1 — ELECTRONIC SUPPLEMENTARY MATERIAL [file 13244_2024_1783_MOESM1_ESM.pdf]
